# Pancreatic hamartoma presenting with sleep-related behavioral abnormalities due to neuroglycopenia: a case report

**DOI:** 10.3389/fonc.2026.1959029

**Published:** 2026-10-01

**Authors:** Xin Dai, Lingchun Liu, Xueye Mao, Shan Li, Yixin Su, Qiang Meng

**Affiliations:** 1 Department of Neurology, The First People’s Hospital of Yunnan Province, Kunming, China; 2 Medical School,Kunming University of Science and Technology, Kunming, China

**Keywords:** case report, hyperinsulinemic hypoglycemia, neuroglycopenia, pancreatic hamartoma, sleep-related behavioral disorder

## Abstract

Pancreatic hamartoma (PH) is an exceedingly rare benign non-neoplastic lesion of the pancreas. Owing to its nonspecific clinical and radiological features, it is prone to misdiagnosis. Among its various subtypes, functionally active pancreatic hamartomas with distinct endocrine activity have only been sporadically reported in the literature as individual cases. We report the case of a 58-year-old female patient whose initial presentation was characterized by paroxysmal behavioral abnormalities during nocturnal sleep, manifesting as sudden agitation, arm-flinging, and purposeless stereotyped movements after sleep onset. During these episodes, the patient exhibited a confusional state with subsequent complete amnesia for the events, a clinical phenotype highly suggestive of REM sleep behavior disorder (RBD) or frontal lobe epilepsy. In-hospital monitoring revealed refractory nocturnal hypoglycemia. Imaging studies identified a mass in the pancreatic tail, and the patient underwent laparoscopic distal pancreatectomy. Postoperative histopathological and immunohistochemical examination confirmed the diagnosis of pancreatic hamartoma, composed predominantly of islet cells (approximately 90%) with associated islet cell tumor-like hyperplasia. Following surgery, the patient’s nocturnal hypoglycemia and sleep-related behavioral abnormalities resolved completely.

## Introduction

Hamartoma is defined as a non-neoplastic, non-progressive space-occupying lesion formed by the aberrant arrangement and disproportionate proliferation of mature, normal tissue components during organ development; it is not a true neoplasm (1). Pancreatic hamartomas are extremely uncommon, accounting for less than 1% of all hamartomas (2). They predominantly occur in the pancreatic head, lack specific clinical manifestations, and are usually non-functional. Common symptoms include upper abdominal pain, discomfort, and bloating, while a considerable number of patients are asymptomatic and diagnosed incidentally during routine check-ups. Radiologically, PH typically presents as a well-defined solid or mixed cystic-solid mass with variable enhancement on contrast-enhanced scans, making preoperative differentiation from pancreatic neuroendocrine tumors (PNETs), solid pseudopapillary neoplasms, or even pancreatic adenocarcinoma challenging (3). Definitive diagnosis relies on postoperative histopathological and immunohistochemical examination.

This report describes a unique case of pancreatic hamartoma presenting with sleep-related abnormal behaviors as the initial manifestation. We aim to highlight the clinical-pathophysiological correlation between a benign islet-predominant pancreatic hamartoma and neuroglycopenia, and to enhance clinical awareness of atypical presentations of functional pancreatic lesions.

## Case description

A 58-year-old female office worker was admitted to the Department of Neurology at the First People’s Hospital of Yunnan Province on June 12, 2026, with a six-month history of nocturnal behavioral abnormalities. The episodes, attributed to chronic work-related stress, occurred repeatedly between 4:00 AM and 6:00 AM and were characterized by behaviors such as folding blankets, standing and walking, wiping tables, shouting, repeatedly touching her head, and thrashing in bed. These episodes were accompanied by urinary incontinence. Family members’ attempts to arouse her during the episodes were ineffective, and she frequently fell out of bed. The patient experienced no choking or awakening during the episodes and had complete amnesia for the events upon waking. She had significant snoring with a loud volume, and family members reported observed apnea episodes. She typically woke spontaneously around 7:00–8:00 AM and felt normal afterward. Since symptom onset, she reported decreased energy and concentration, with a weight loss of approximately 5 kg over the preceding six months.

The patient had a 10-year history of hypertension, managed with regular oral irbesartan. She had been diagnosed with moderate obstructive sleep apnea syndrome one month prior but had not received specific treatment. Family members reported multiple episodes of nocturnal hypoglycemia, with documented blood glucose levels around 2.0 mmol/L. She denied any history of diabetes mellitus, prior surgeries, trauma, or allergies.

Vital signs: temperature 36.5 °C, pulse 83 beats/min, respiratory rate 20/min, blood pressure 142/87 mmHg, BMI 19.5 kg/m². She was alert, with coherent speech and cooperative during examination. Neurological examination: orientation, memory, calculation, and judgment were normal. Speech was fluent with clear articulation. Cranial nerve examination was unremarkable. Muscle strength was grade 5/5 in all limbs, with normal tone and symmetric deep tendon reflexes. Pathological reflexes were absent. Superficial and deep sensation, as well as coordination, were normal. Cardiopulmonary and abdominal examinations revealed no significant abnormalities.

Laboratory Findings: In-hospital nocturnal glucose monitoring revealed frequent hypoglycemic episodes, with levels dropping as low as 1.2–2.0 mmol/L. Concurrent testing demonstrated inappropriately elevated insulin levels: serum C-peptide (fasting) 2.32 ng/mL, insulin (fasting) 9.96 μU/mL, with a significantly elevated insulin-to-glucose ratio, consistent with hyperinsulinemic hypoglycemia. An oral glucose tolerance test (OGTT) showed a fasting glucose of 3.8 mmol/L and a 2-hour postprandial glucose of 14.4 mmol/L, indicating impaired glucose tolerance. Serum electrolytes, renal and hepatic function, thyroid function, cortisol rhythm, ACTH, and tumor markers were all within normal limits.

Neurophysiological Examination: Electroencephalography (EEG) performed after admission revealed no definite epileptiform discharges.

Imaging Studies: MRI of the hepatobiliary system and pancreas (plain and contrast-enhanced, performed on June 16, 2026) demonstrated a well-defined, approximately 1.9 cm round lesion in the inferior aspect of the pancreatic tail, exhibiting heterogeneous signal with slightly prolonged T1 and T2. On thin-slice dynamic contrast-enhanced imaging, the lesion showed heterogeneous enhancement with prominent peripheral enhancement and a close relationship with the splenic vein, appearing to connect with the pancreatic tail (**Figure 1**). Although this peripheral enhancement pattern was noted, it did not allow a definitive diagnosis or reliable differentiation from a neuroendocrine tumor, a solid pancreatic neoplasm, or other pathologies, highlighting that imaging alone was insufficient for preoperative characterization and that surgical exploration was appropriately justified.

**Figure 1:**
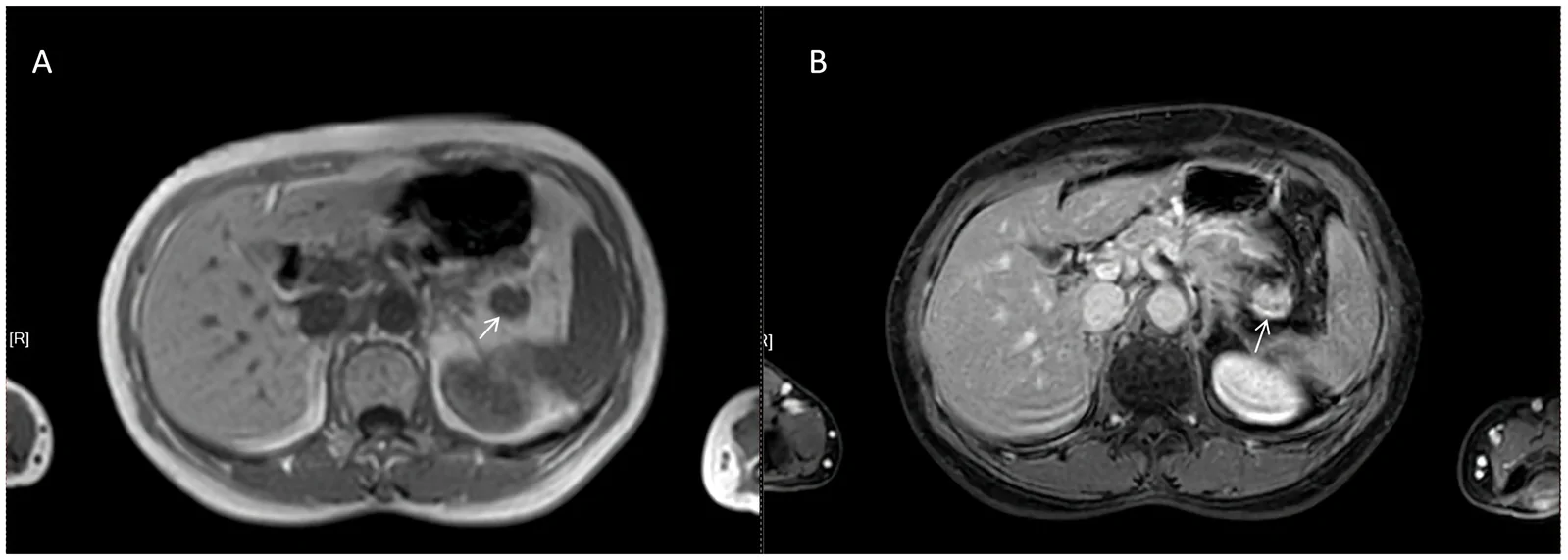
Preoperative MRI of the pancreatic lesion. **(A)** Plain MRI showing a well-defined, approximately 1.9 cm round lesion in the inferior aspect of the pancreatic tail, with heterogeneous signal and slightly prolonged T1 and T2. **(B)** Contrast-enhanced MRI showing heterogeneous enhancement with prominent peripheral enhancement, closely related to the splenic vein and appearing to connect with the pancreatic tail.

Upon admission, the patient was started on a bedtime snack and enteral nutritional support to prevent nocturnal hypoglycemia, with limited effectiveness. The imaging findings, showing a ring-like and progressive enhancement of the nodule between the pancreatic tail and splenic hilum, favored a benign lesion, but an insulinoma could not be excluded. On June 23, 2026, the patient underwent a laparoscopic distal pancreatectomy under general anesthesia. Intraoperatively, a reddish, firm, well-encapsulated tumor approximately 2 cm in size was identified in the pancreatic body-tail region and was completely resected and sent for pathological examination. Postoperative routine histopathology and immunohistochemistry results were as follows:

Gross examination: A 5 cm × 2.5 cm × 2.2 cm pancreatic mass was received, with a 2.5 cm × 1.9 cm × 1.8 cm, well-circumscribed, solid tumor with a gray-white to slightly gray-yellowish cut surface and firm consistency. The lesion is suspicious for a neuroendocrine tumor. No vascular invasion, perineural invasion, or infiltration into the adjacent pancreatic parenchyma is identified (**Figure 2**).

**Figure 2:**
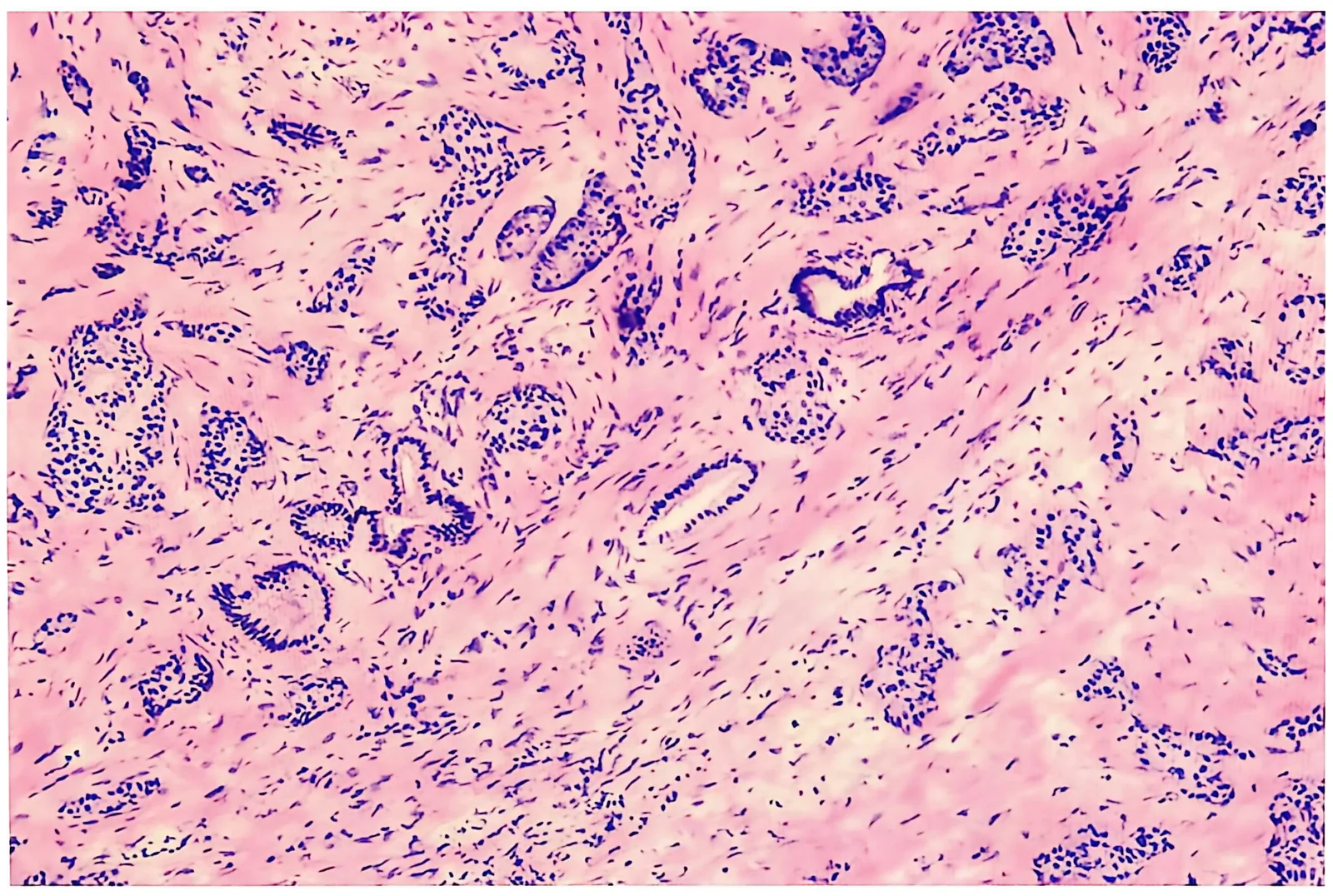
Light microscopy findings of the pancreatic hamartoma (HE staining). Hematoxylin and eosin staining showing a well-circumscribed, unencapsulated tumor composed predominantly of endocrine cells, with scattered ductal structures and small acinar clusters forming a disordered mosaic. No vascular invasion, perineural invasion, or infiltration into the adjacent pancreatic parenchyma is identified.

Immunohistochemistry revealed: PCK (+), Vim (+), CK7 (focal +), CK19 (focal +), CEA (-), P53 (-), Syn (strongly positive in most cells), CD56 (strongly positive in most cells), INSM1 (strongly positive in most cells), Ki-67 (+ with a hotspot of ~2%), CD34 (focal +), CD117 (-), CgA (strongly positive in most cells), with a concentric arrangement pattern (**Figure 3**).

**Figure 3:**
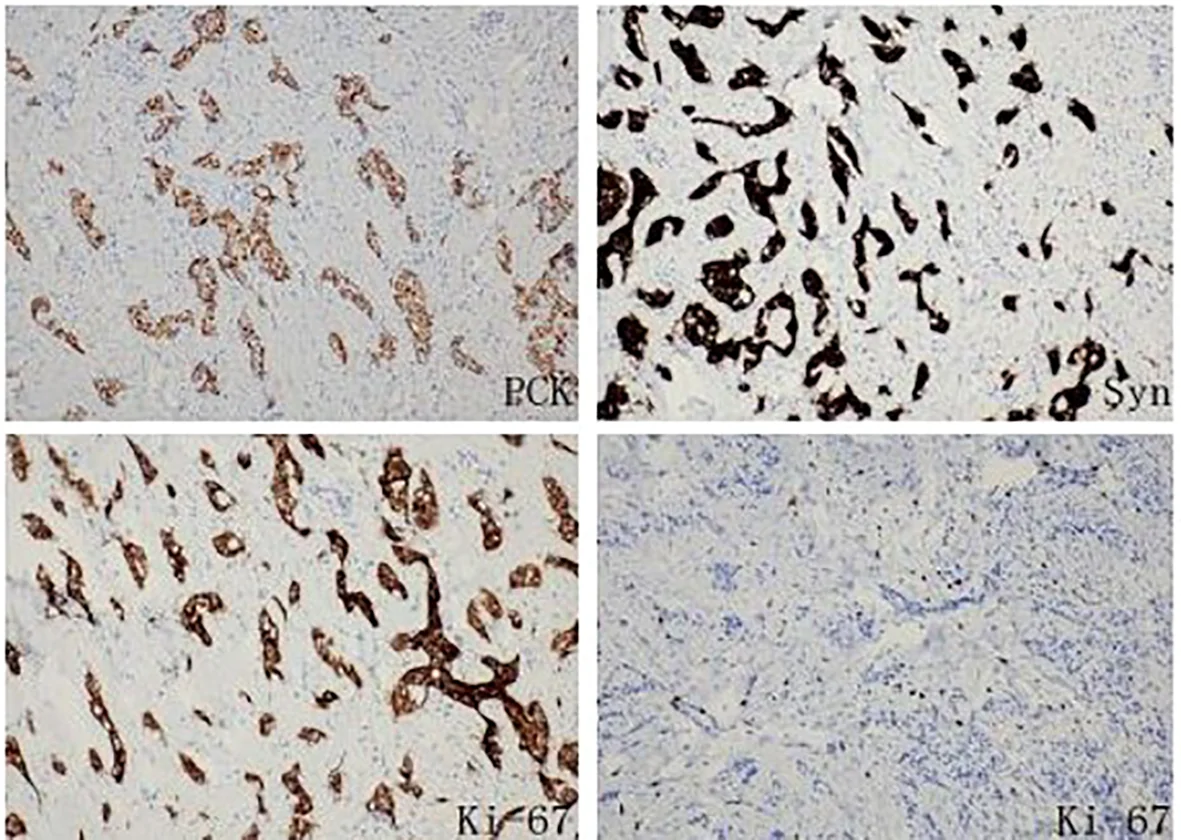
Immunohistochemical findings of the pancreatic hamartoma. Immunohistochemical staining showing positivity for PCK, Vim, CK7 (focal), CK19 (focal), Syn, CD56, INSM1, CD34 (focal), and CgA, with a Ki-67 hotspot of approximately 2% and negative p53, supporting a benign pancreatic hamartoma with marked endocrine predominance.

Pathological Diagnosis: Grossly, the lesion was a well-circumscribed but unencapsulated nodule measuring approximately 2.0 cm. Histologically, it was composed predominantly of endocrine cells (about 90%), but immunohistochemical staining for cytokeratin (ductal) and trypsin (acinar) revealed that these cells were haphazardly intermingled with scattered ductal structures and small acinar clusters, forming a disordered mosaic rather than a clonal endocrine population. The lesion showed a pushing border without perineural or vascular invasion, and elastic van Gieson staining demonstrated no concentric perivascular fibrosis. The endocrine cells were diffusely positive for synaptophysin, CD56, INSM1, and chromogranin A, with a Ki-67 index of only 2% and negative p53 staining. These features support a benign pancreatic hamartoma with marked endocrine predominance rather than an insulinoma or pancreatic neuroendocrine tumor.

Postoperatively, the patient recovered well. Fasting and nocturnal blood glucose levels returned to the normal range, and the sleep-related abnormal behaviors completely resolved. No recurrence of hypoglycemia or sleep-related behavioral episodes was noted during the follow-up period. Given the benign nature of hamartomas, long-term oncological surveillance is not routinely required; nonetheless, we plan to continue clinical and biochemical follow-up (including fasting glucose and insulin levels) at 6 months and 1 year postoperatively to ensure sustained remission and to monitor for any late recurrence.

## Timeline of care

The complete clinical course is summarized in the **Figure 4**.

**Figure 4:**
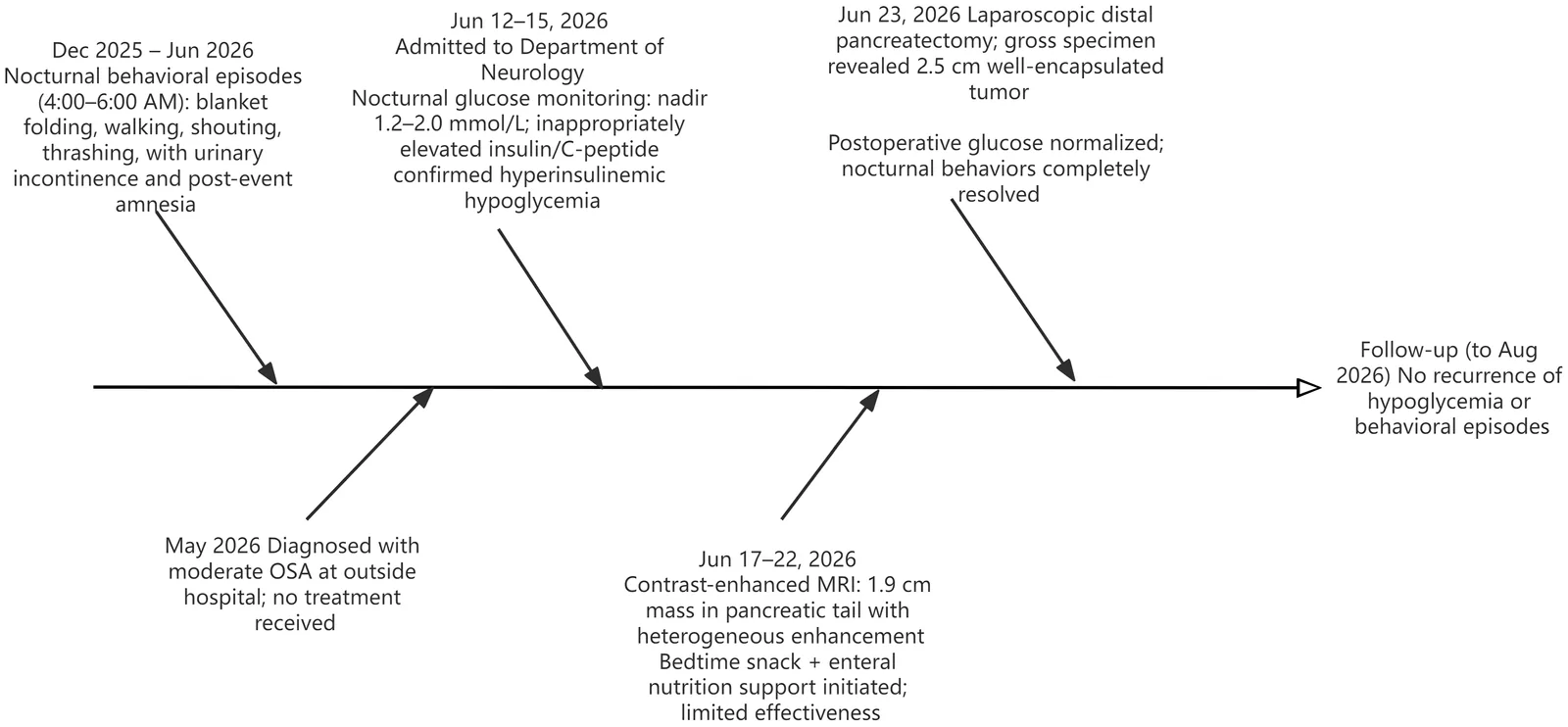
Timeline of the patient’s clinical course. The timeline summarizes the onset of nocturnal behavioral episodes, admission to the Department of Neurology, diagnostic evaluation, initiation of nutritional support, laparoscopic distal pancreatectomy, postoperative resolution of hypoglycemia and behavioral abnormalities, and follow-up.

### Diagnostic assessment

Diagnostic methods and rationale: Nocturnal glucose monitoring revealed recurrent hypoglycemia (nadir 1.2–2.0 mmol/L) with inappropriately elevated insulin (9.96 μU/mL) and C-peptide (2.32 ng/mL), confirming hyperinsulinemic hypoglycemia. OGTT showed impaired glucose tolerance (fasting 3.8 mmol/L, 2-hour 14.4 mmol/L). EEG excluded epileptiform discharges. Contrast-enhanced MRI identified a 1.9 cm well-defined lesion in the pancreatic tail with heterogeneous enhancement.

Diagnostic challenges: The neurological presentation initially suggested a primary sleep or seizure disorder; non-specific imaging features could not definitively distinguish PH from insulinoma preoperatively.

Differential diagnosis: Insulinoma, functional pancreatic hamartoma, RBD, nocturnal frontal lobe epilepsy, and other causes of neuroglycopenia.

Intervention type and rationale: Laparoscopic distal pancreatectomy was performed based on: (1) a functional pancreatic mass causing severe refractory hypoglycemia; (2) failure of conservative measures; and (3) need for definitive diagnosis and curative treatment.

Intervention details: The 2 cm reddish, firm, well-encapsulated tumor in the pancreatic body-tail was completely resected under general anesthesia.

Modifications and adverse events: No intraoperative or postoperative complications occurred.

### Follow-up and outcomes

Clinician-assessed outcomes: Postoperative fasting and nocturnal glucose normalized; hypoglycemic episodes ceased; sleep-related behavioral abnormalities completely resolved.

Pathological outcomes: Immunohistochemistry confirmed pancreatic hamartoma composed predominantly of islet cells (~90%) with islet cell tumor-like hyperplasia (Syn+, CD56+, CgA+, Ki-67 ~2%).

Patient-reported outcomes: The patient reported improved energy, concentration, and sleep quality, with high satisfaction.

Adverse events: None observed during follow-up.

## Discussion

Pancreatic hamartoma is a non-neoplastic developmental malformation composed of mature, differentiated pancreatic tissue components that are abnormally arranged and disproportionately proliferated. By definition, it is not a true neoplasm but a structural anomaly arising during embryogenesis (4). This entity is extremely rare, first described by Anthony et al. in 1977 (5), and accounts for less than 1% of all pancreatic tumor-like lesions. Histologically, PH is characterized by the irregular, haphazard distribution of acinar, ductal, and islet cells within a fibrous stroma, lacking the normal pancreatic lobular architecture and the hierarchical branching of ducts and acini. Most previously reported cases presented with abdominal pain, discomfort, or were incidental findings (6); functional endocrine abnormalities are exceptionally rare, with only a few scattered reports (7, 8). In the present case, the hamartoma consisted of approximately 90% islet cells, granting the lesion functional activity akin to that of an islet cell tumor. This resulted in the continuous, dysregulated secretion of insulin from the abundant islet tissue, leading to hyperinsulinemia and refractory hypoglycemia.For surgical oncologists, the key take-home message of this case is that any functioning pancreatic mass causing refractory hypoglycemia—even in the absence of classic imaging features of insulinoma—warrants serious surgical consideration, as benign hamartomas can produce the same severe clinical syndrome as malignant or neuroendocrine tumors.

Under physiological conditions, insulin secretion from pancreatic β-cells is tightly regulated by a negative feedback mechanism sensitive to blood glucose concentrations. However, within the hamartoma, the large population of islet cells is detached from the normal pancreatic lobular structure and is arranged haphazardly, losing normal glucose sensing and feedback inhibition. This leads to the “autonomous” secretion of insulin, which is not suppressed by hypoglycemia. Insulinoma, the most common functional PNET, induces hypoglycemia through a similar mechanism, where neoplastic β-cells secrete excessive insulin, suppressing hepatic glucose output and causing fasting hypoglycemia (9). An additional noteworthy laboratory finding in this case was the oral glucose tolerance test (OGTT). It showed a fasting glucose of 3.8 mmol/L and a 2-hour value of 14.4 mmol/L.

This pattern is actually consistent with the pathophysiology of autonomous insulin secretion. The fasting glucose remained at the low-normal range because the patient was in a relatively steady state overnight. When challenged with a 75g glucose load, the rapid influx of exogenous glucose, combined with dysregulated insulin release from the tumor, produced an exaggerated postprandial hyperglycemic spike. At the same time, the patient’s residual normal β-cells may have been chronically suppressed by the hyperinsulinemic environment. This contributed to a sluggish and insufficient early-phase insulin response to the oral load. This biphasic disturbance has been described in insulinoma and other islet cell hyperfunction syndromes. It indicates that the patient had profound disruption of glucose homeostasis in both the fasting and fed states.

The core mechanism underlying the sleep-related behavioral abnormalities is neuroglycopenia (10). Glucose is almost the sole energy substrate for brain cells. Since the brain cannot synthesize glucose or store significant glycogen, its energy supply is entirely dependent on a continuous blood glucose supply. When excessive insulin secretion from the hamartoma drives blood glucose levels below 2.8 mmol/L, cerebral energy metabolism becomes impaired, leading to progressive cortical dysfunction (11). Symptoms typically begin with the cerebral cortex, manifesting as poor concentration, confusion, and bizarre behavior (12). As hypoglycemia worsens, dysfunction may extend to subcortical structures, the midbrain, and even the medulla, resulting in confusion, seizures, coma, hemiplegia (13), or even transient interhemispheric disconnection (14). In this case, with blood glucose levels reaching as low as 1.2 mmol/L, the severe hypoglycemic state led to significant cortical impairment, manifesting as complex sleep-related abnormal behaviors, including purposeless complex motor behaviors (folding blankets, walking, wiping tables, touching head) and retrograde amnesia with complete inability to recall the events.

The episodes displayed a striking temporal specificity, occurring predominantly between 4:00 AM and 6:00 AM, which corresponds to the later stages of the sleep cycle. The combination of fasting-induced suppression of endogenous glucose production and sleep-related low sympathetic tone likely constitutes the pathophysiological basis for the nocturnal timing of these neuroglycopenic episodes. Similar temporal associations have been reported in the literature. Suzuki et al. (15) described a 65-year-old patient with insulinoma who exhibited sudden laughing, clapping, and bizarre posturing in the early morning, with blood glucose dropping to 0.83 mmol/L. Yang et al. (16) reported a 79-year-old patient with type 2 diabetes who presented with lower limb jerking, searching movements, and crawling with eyes closed around 4:30 AM daily, with a blood glucose of 35 mg/dL. Concurrent EEG demonstrated diffuse theta and delta activity without epileptiform discharges, which rapidly resolved upon intravenous glucose administration.

This case report describes a functional pancreatic hamartoma that manifested initially with symptoms resembling a sleep behavior disorder, with the underlying pathophysiology being neuroglycopenia secondary to hyperinsulinemic hypoglycemia. For patients presenting with recurrent, early-morning episodes of confusional states accompanied by complex motor behaviors, particularly when associated with fasting hypoglycemia, clinicians should broaden the differential diagnosis beyond primary neurological conditions such as RBD or frontal lobe epilepsy. Hyperinsulinemic hypoglycemia should be considered as a key differential, and while investigating radiologically, one must not only focus on insulinoma but also remain vigilant for the possibility of a functional pancreatic hamartoma.

In summary, the pathogenesis of functional pancreatic hamartomas remains unclear. In the present case, the tumor contained about 90% islet cells. This most likely explains the patient’s hyperinsulinemic hypoglycemia. However, why this hamartoma developed such a dominant islet-cell population is unknown. This report has several limitations. It is a single case with a descriptive design. And the link between the hamartoma and the sleep-related behaviors is based on clinical observation. The episodes resolved after surgery, but we did not obtain direct mechanistic evidence to prove causality. Our follow-up period is short. Long-term outcomes beyond one year are not yet available. Given that current knowledge relies almost exclusively on individual case reports, larger studies are needed. Multicenter case series with standardized data collection would help to better define the clinical spectrum of functional pancreatic hamartomas and guide clinical decisions.

## Patient perspective

“I had been suffering from these strange nighttime episodes for nearly six months. My family was terrified—they didn’t know what was happening to me, and neither did I, because I could never remember anything in the morning. I was worried I might have a brain problem or epilepsy. The doctors in the neurology department were very thorough. They checked my blood sugar at night and found it was dangerously low, which surprised all of us. When they told me there was a mass in my pancreas and that surgery might cure me, I was anxious but also hopeful. The surgery went smoothly, and from the very first night after the operation, the episodes stopped. I feel like I have gotten my life back. I can sleep peacefully now, and my family no longer has to watch over me in fear. I am grateful to the medical team for not giving up on finding the real cause of my problem.”

## Data Availability

The original contributions presented in the study are included in the article/supplementary material. Further inquiries can be directed to the corresponding author.
